# Oxycodone alleviates mifepristone‐stimulated human endometrial stromal cell injury by activating the Keap1/Nrf2/HO‐1 signaling pathway

**DOI:** 10.1002/iid3.1008

**Published:** 2023-09-20

**Authors:** Aibing Zhu, Fei Yao, Mingkun Shen

**Affiliations:** ^1^ Department of Anesthesiology Wuxi Maternity and Child Health Care Hospital Wuxi China

**Keywords:** endometrial injury, Keap1/Nrf2/HO‐1, oxycodone

## Abstract

**Background:**

Endometrial injury is a common disease in women caused by intrauterine inflammation, infections, and endocrine disorders. Human endometrial stromal cells (hEndoSCs) can maintain endometrial homeostasis and play an important role in repairing endometrial injury. Mifepristone, a steroidal anti‐progesterone drug, is widely used in the field of reproductive medicine worldwide. Mifepristone‐induced hEndoSC injury has been used to study endometrial injury in vitro. At present, the pathogenesis and potential regulatory mechanisms of oxycodone in endometrial injury remain unknown.

**Aims:**

We aimed to evaluate the functions of oxycodone in mifepristone‐stimulated hEndoSC injury and analyze its potential molecular mechanism.

**Materials & Methods:**

hEndoSC viability, cytotoxicity, and apoptosis were analyzed using the methyl thiazolyl tetrazolium assay, the lactate dehydrogenase assay, and flow cytometry, respectively. Furthermore, the levels of cleaved‐Caspase3, Keap1, Nrf2, HO‐1, and NQO1 were assessed using reverse transcription quantitative polymerase chain reaction and western blot analysis, and the release of inflammatory cytokines was determined using the enzyme‐linked immunosorbent assay.

**Results:**

We observed that oxycodone had no adverse effects on hEndoSCs; rather, it protected hEndoSCs against mifepristone‐induced endometrial damage, as confirmed by the enhanced cell viability, reduced number of apoptotic cells, decreased Caspase3 activity and inflammatory cytokine secretion, and increased Keap1/Nrf2/HO‐1 pathway‐related protein expression. In addition, we found that the protective effects of oxycodone on mifepristone‐induced hEndoSC injury were inhibited by ML385 (a Keap1/Nrf2/HO‐1 inhibitor).

**Conclusion:**

In summary, we confirmed that oxycodone alleviates mifepristone‐induced hEndoSC injury by activating the Keap1/Nrf2/HO‐1 signaling pathway.

## Graphical abstract

The present study explores the effects of oxycodone on mifepristone‐induced hEndoSC injury and explains the underlying molecular mechanism. The findings revealed that oxycodone alleviated mifepristone‐triggered endometrial injury by activating the Keap1/Nrf2/HO‐1 signaling pathway, which may provide a theoretical basis for endometrial injury treatment.

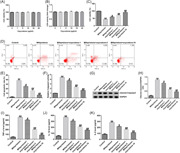

## 1 INTRODUCTION

Endometrial injury is a disease that causes local morphological trauma and functional disorders of the endometrium due to physiological factors such as uterine position, uterine cavity compatibility and constitution, or pathological factors such as mechanical injury, local trauma infection, and endocrine immune disorders.^1^, ^2^ The main symptoms of endometrial damage include dysmenorrhea, menstrual disorders, and endometriosis.^3^, ^4^ Endometrial injury is a major cause of adverse pregnancy outcomes such as female infertility and spontaneous abortion.^5^, ^6^ Therefore, an in‐depth study of the pathogenesis of endometrial injury and the identification of new therapeutic targets are key to improving the therapeutic effect of endometrial injury.

Oxycodone is a semisynthetic opioid derivative that has good analgesic effects. It has been reported that oxycodone improves gastrointestinal function under effective analgesia.^7^ In addition, oxycodone is widely used in clinics due to its high bioavailability and multiple routes of administration.^8^ Oxycodone combined with opioid receptor antagonists can also reduce opioid‐induced constipation symptoms.^9^ Green et al.^10^ revealed that maternal oxycodone treatment led to changes in mouse placental histology. A recent research reported that oxycodone alleviates endometrial injury by inhibiting the activation of the TLR4/NF‐kB pathway.^11^ However, the underlying molecular mechanisms under the effect of oxycodone against endometrial injury remain to be explored.

Multiple factors are associated with the progression of endometrial diseases.^12^, ^13^, ^14^ In addition, the Keap1/Nrf2/HO‐1 pathway has been implicated in several diseases.^15^, ^16^ Zborowski et al.^15^ suggested that the Keap1/Nrf2/HO‐1 signaling pathway contributes to the antidepressant‐like action of *p*‐chlorodiphenyl diselenide in diabetic mice.^15^ In addition, research has shown that the Keap1/Nrf2/HO‐1 signaling pathway plays a crucial role in the regulation of apoptosis, oxidative stress, and inflammation in tissue damage.^17^, ^18^ However, whether the Keap1/Nrf2/HO‐1 signaling pathway is associated with the progression of endometrial injury needs to be elucidated further.

The human endometrial stromal cells (hEndoSCs) are responsible for vascular reconstruction, endometrial decidualization, immune cell recruitment, and large molecule production, which can maintain endometrial homeostasis and play an important role in repairing endometrial injury.^19^ Endometrial damage may lead to hEndoSC apoptosis and decreased hEndoSCs activity, leading to endometrial atrophy and failure of endometrial homeostasis.^20^ Mifepristone is a steroidal antiprogesterone drug, and it is widely used around the globe in the field of reproductive medicine.^21^ Mifepristone‐induced damage of hEndoSCs has been confirmed on the basis of molecular, morphological, and functional features.^11^, ^22^ In recent years, mifepristone‐induced hEndoSCs have been used as an in vitro‐induced endometrial injury model.^11^, ^23^, ^24^ In this study, we will use mifepristone‐induced hEndoSCs to investigate the effect of oxycodone on endometrial damage.

Therefore, this study aimed to illustrate the function of oxycodone in mifepristone‐stimulated hEndoSCs during the development of endometrial injury. In our study, we hypothesized that (i) hEndoSCs exposed to mifepristone could be used to establish an endometrial injury model, (ii) oxycodone alleviates mifepristone‐induced endometrial injury in hEndoSCs, and (iii) the underlying mechanisms of the protective effects of oxycodone may be related to the activation of the Keap1/Nrf2/HO‐1 pathway.

## 2 MATERIALS AND METHODS

### 2.1 Establishment of the endometrial injury model

hEndoSCs were obtained from Procell and maintained in Dulbecco's modified Eagle's medium (DMEM) (Invitrogen) containing 10% fetal bovine serum (Gibco), 100 mg/mL streptomycin, and 100 IU/mL penicillin in a humidified incubator with 5% CO_2_ at 37°C. Next, mifepristone (60 μmol/L, 48 h) was used to stimulate hEndoSCs to establish an endometrial injury model.^24^

For oxycodone treatment, hEndoSCs were exposed to 1, 5, 10, 15, or 20 μg/mL oxycodone for 24 h.^11^

To explore whether the Keap1/Nrf2/HO‐1 signaling pathway was involved in the action of oxycodone on mifepristone‐induced hEndoSCs, a Keap1/Nrf2/HO‐1 pathway inhibitor (ML385) was used. Also, the mifepristone‐induced hEndoSCs were treated with oxycodone (10 μg/mL) or/and 5 μM ML385 for 24 h.

### 2.2 Methyl thiazolyl tetrazolium (MTT) assay

After treatment, hEndoSCs were cultured in 96‐well plates at 37°C. Approximately 20 μL of MTT (Sigma‐Aldrich) was then added to each well and incubated for 4 h. Next, the culture medium was removed and cells were dissolved in 150 μL of dimethyl sulfoxide (DMSO, Sigma‐Aldrich) under dark conditions for 10 min. Finally, the optical density (OD) was measured at 570 nm using a microplate reader (Bio‐Rad) according to the manufacturer's protocol.

### 2.3 Flow cytometry analysis

To detect apoptosis, the hEndoSCs were treated with oxycodone. Cells were subsequently washed, centrifuged, and harvested. The cells were then incubated with Annexin V‐fluorescein isothiocyanate/propidium iodide (Beyotime) for 30 min at 37°C under dark conditions according to the manufacturer's protocol. Finally, apoptotic cells were quantified and analyzed using a flow cytometer (BD Biosciences).

### 2.4 Detection of caspase3 activity

caspase3 activity in hEndoSCs was determined using a caspase3 Assay Kit (Abcam) following the manufacturer's instructions. Briefly, the hEndoSCs were lysed, centrifuged, and collected. The supernatant was then treated with caspase3 reagent, and the OD_405nm_ value was determined using a microplate reader (Thermo Fisher Scientific).

### 2.5 Western blot assay

After treatment, hEndoSC proteins were collected using radioimmunoprecipitation lysis buffer (Beyotime). Bicinchoninic acid protein quantitative kits (Solarbio) were used to measure the protein concentration according to the manufacturer's protocol. The extracted proteins were resolved using 10% sodium dodecyl sulfate‐polyacrylamide gel electrophoresis and transferred onto a polyvinylidene fluoride membrane. Membranes were blocked with 5% skim milk at room temperature for 1 h and incubated with primary antibodies (cleaved‐Caspase3, Keap1, Nrf2, HO‐1, NQO1, and GAPDH) at a dilution of 1:1000 overnight at 4°C. Membranes were then incubated with secondary antibodies for 1 h. Finally, the protein bands were visualized using electrochemiluminescence (ECL) detection system reagents (Pierce) and quantified using ImageJ software.

### 2.6 Enzyme‐linked immunosorbent assay (ELISA) assay

After treatment, the secretion of tumor necrosis factor alpha (TNF‐α), interleukin‐β (IL‐β), and IL‐6 in the supernatant of hEndoSCs was detected using ELISA kits following the manufacturer's protocol.

### 2.7 Lactate dehydrogenase (LDH) assay

LDH released from hEndoSCs was determined using an LDH‐Cytotoxicity Assay kit (Sigma‐Aldrich). hEndoSCs were seeded in 12‐well plates and grown for 48 h. After treatment, hEndoSC supernatants were collected from each well and the cell lysates were incubated with the LDH reaction mixture for 15 min according to the manufacturer's instructions. Absorbance was measured at 490 nm using a microplate reader (BioTek).

### 2.8 Reverse transcription quantitative polymerase chain reaction (RT‐qPCR) analysis

After treatment, the levels of Keap1, Nrf2, HO‐1, NQO1, and β‐actin were measured using RT‐qPCR. RNA was isolated from hEndoSCs using an RNA isolation kit (Life Technologies Corporation) as per the manufacturer's protocol. Next, the total RNA was reversed to cDNA according to the protocols of the cDNA Synthesis Kit (TaKaRa) and RT‐qPCR analysis was conducted using the SYBR Green Master mix (TaKaRa) and the ABI 7500 Real‐Time PCR System (Agilent Technologies). Gene expression was determined using the $2^{-\Delta \Delta C_{t}}$ method. Primer sequences for PCR were as follows:

Keap1 forward 5′‐ CCAATGCTGACACGAAGGAT‐3′;

Reverse 5′‐ATACAGTTGTGCAGGACGCAG‐3′.

Nrf2 forward 5′‐CAGCACATCCAGACAGACACCAG‐3′;

Reverse 5′‐GGCAAGCGACTCATGGTCATCTAC‐3′.

HO‐1 forward 5′‐AGCGAAACAAGCAGAACCCAGTC‐3′;

Reverse 5′‐GCTGTGTGGCTGGTGTGTAAGG‐3′.

NQO1 forward 5′‐GAAGAGCACTGATCGTACTGGC‐3′;

Reverse 5′‐GGATACTGAAAGTTCGCAGGG‐3′.

β‐actin forward 5′‐TCACCCACACTGTGCCCATCTACGA‐3′;

Reverse 5′‐CAGCGGAACCGCTCATTGCCAATGG‐3′.

### 2.9 Statistical analysis

All experiments were performed three times. All data were presented as the means ± SD from three independent experiments and analyzed using SPSS 19.0 (SPSS Inc.). Statistically significant differences among multiple groups were estimated using one‐way analysis of variance (ANOVA), followed by Tukey's post hoc test. *p < .05 was considered to indicate statistical significance.

## 3 RESULTS

### 3.1 Oxycodone had no cytotoxic effect on hEndoSCs

**Figure 1:**
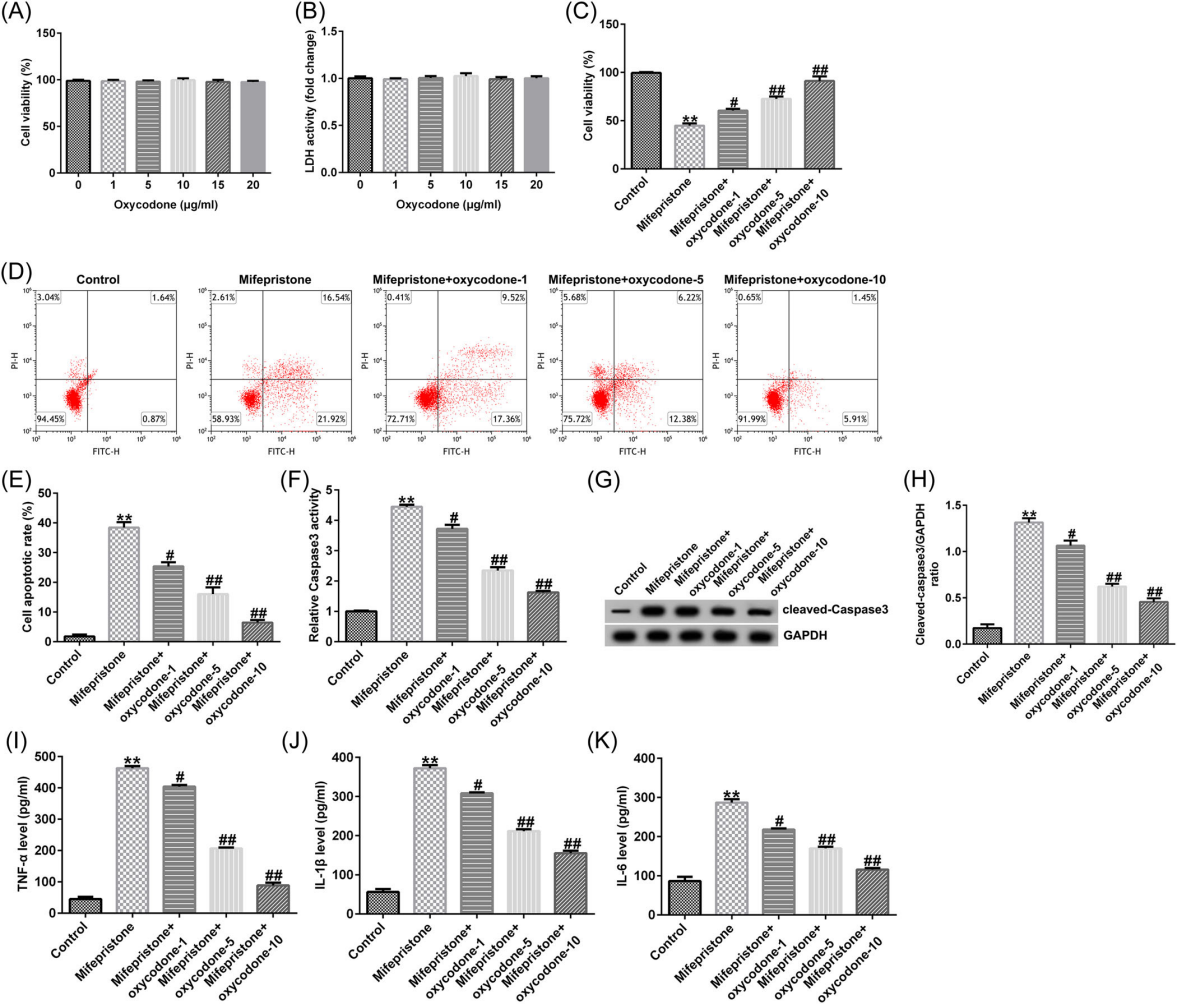
Influence of oxycodone on mifepristone‐induced human endometrial stromal cell (hEndoSC) damage. hEndoSCs were exposed to various concentrations of oxycodone (1, 5, 10, 15, or 20 μg/mL) for the indicated times. (A) Methyl thiazolyl tetrazolium (MTT) assay for cell viability. (B) Cell injury was assessed by lactate dehydrogenase (LDH) release. hEndoSCs were treated with mifepristone, followed by treatment with 1, 5, or 10 μg/mL oxycodone for 24 h. (C) The viability of hEndoSCs was detected using the MTT assay. (D) Apoptosis of hEndoSCs was determined using flow cytometry (FCM). (E) Quantification of apoptotic cells. (F) Detection of Caspase3 activity. (G) Western blot analysis of cleaved‐Caspase3. (H) Quantification of cleaved‐Caspase3 expression. The enzyme‐linked immunosorbent assay was applied to assess tumor necrosis factor‐α (TNF‐α), interleukin‐β (IL‐β), and IL‐6 (I–K) expression in the different groups. *N* = 3. **p < .01 versus Control; ^#, ##^ *p* < .05, .01 versus the mifepristone group. Experiments were repeated three times, and the data were presented as means ± SD.

We investigated the effect of oxycodone on mifepristone‐induced endometrial damage. hEndoSCs were exposed to 1, 5, 10, 15, or 20 μg/mL oxycodone ^11^ for different time periods. MTT and LDH leakage assays were conducted to assess cell viability and LDH secretion in different groups, respectively. We found that there were no significant changes in cell viability and LDH release in the oxycodone groups compared with the control group (Figure 1A,B), suggesting that oxycodone had no cytotoxic effect on hEndoSCs.

### 3.2 Oxycodone reversed the effects of mifepristone on cell viability, cell apoptosis, and Caspase3 activity in hEndoSCs

Mifepristone (60 μmol/L, 48 h) was used to stimulate hEndoSCs to establish an endometrial injury model. The cell morphology of hEndoSCs of the control group and the mifepristone treatment group is presented in Supporting Information: Figure 1. We further assessed the effects of oxycodone in mifepristone‐induced hEndoSCs. hEndoSCs exposed to mifepristone were treated with 1, 5, or 10 μg/mL oxycodone for a specific time. Results from the MTT and flow cytometry analyses suggested that mifepristone inhibited hEndoSC viability (Figure 1C), promoted apoptosis (Figure 1D,E), and enhanced the activity of caspase‐3 (Figure 1F) and cleaved‐Caspase3 expression (Figure 1G,H). Nevertheless, we observed opposite effects in the mifepristone + oxycodone group, demonstrating the protective effect of oxycodone on mifepristone‐induced hEndoSC damage.

### 3.3 Oxycodone reversed the effects of mifepristone on the production of inflammatory cytokines in hEndoSCs

Inflammatory responses have been found to be associated with organ damage after hEndoSC injury. Therefore, we investigated whether the secretion of inflammatory factors, TNF‐α, IL‐β, and IL‐6, was regulated by oxycodone. As shown in Figure 1I–K, mifepristone promoted the release of inflammatory cytokines (TNF‐α, IL‐β, IL‐6) when compared to the control. However, oxycodone suppressed the production of inflammatory cytokines by hEndoSCs in a dose‐dependent manner. Our observations suggest that oxycodone inhibits the release of inflammatory cytokines in mifepristone‐treated hEndoSCs.

### 3.4 Oxycodone regulated the activation of the Keap1/Nrf2/HO‐1 signaling pathway in mifepristone‐stimulated hEndoSCs

**Figure 2:**
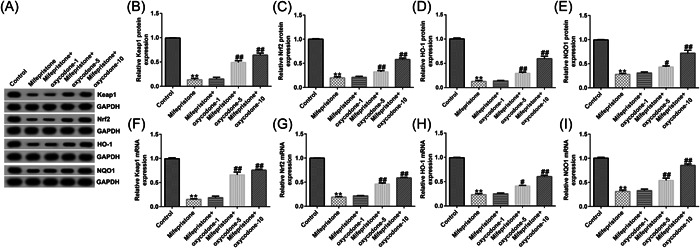
Influence of oxycodone on the mifepristone‐mediated regulation of the Keap1/Nrf2/HO‐1 signal pathway in human endometrial stromal cells (hEndoSCs). hEndoSCs were treated with mifepristone, followed by treatment with 1, 5, or 10 μg/mL oxycodone for different time periods. (A) The protein expression of Keap1, Nrf2, HO‐1, and NQO1 was assessed by western blot analysis. (B–E) Quantification of Keap1, Nrf2, HO‐1, and NQO1. Reverse transcriptase quantitative polymerase chain reaction analysis of Keap1 (F), Nrf2 (G), HO‐1 (H), and NQO1 (I) levels. *N* = 3. **p < .01 versus Control; ^#, ##^ *p* < .05, .01 versus the mifepristone group. Experiments were repeated three times, and the data were presented as means ± SD.

We further aimed to explore the molecular mechanism of oxycodone in mifepristone‐stimulated hEndoSCs. The results suggested that mifepristone treatment markedly suppressed the protein (Figure 2A–E) and mRNA (Figure 2F–I) levels of Keap1, Nrf2, HO‐1, and NQO1 (Figure 2A–E). However, oxycodone reversed the effects of mifepristone on the regulation of the Keap1/Nrf2/HO‐1 signaling pathway, indicating that oxycodone may activate the Keap1/Nrf2/HO‐1 signaling pathway in the mifepristone‐induced endometrial injury model.

### 3.5 ML385 reversed the role of oxycodone in the Keap1/Nrf2/HO‐1 signaling pathway

**Figure 3:**
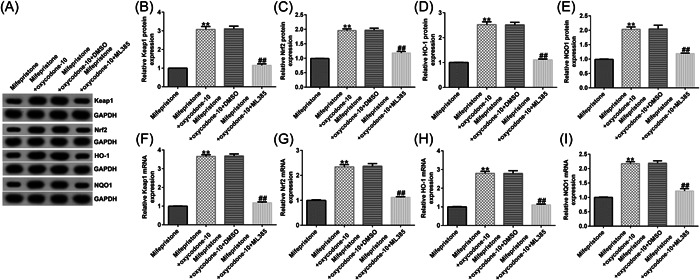
Oxycodone activated the Keap1/Nrf2/HO‐1 signaling pathway and ML385 reversed the effects. Human endometrial stromal cells (hEndoSCs) were exposed to mifepristone for 48 h, and then treated with oxycodone (10 μg/mL) and/or a Keap1/Nrf2/HO‐1 inhibitor (5 μM ML385) for 24 h. Cells were divided into four groups: mifepristone, mifepristone + oxycodone‐10, mifepristone + oxycodone‐10 + DMSO, or mifepristone + oxycodone‐10 + ML385. (A) Detection of Keap1, Nrf2, HO‐1, and NQO1 expression by western blot analysis. (B–E) Statistical analysis of Keap1, Nrf2, HO‐1, and NQO1 levels. Reverse transcription quantitative polymerase chain reaction analysis of Keap1 (F), Nrf2 (G), HO‐1 (H), and NQO1 (I) levels. *N* = 3. **p < .01 versus mifepristone; ^##^ *p* < .01 versus the mifepristone + oxycodone‐10 + DMSO group. Experiments were repeated three times, and the data were presented as means ± SD.

Next, we aimed to investigate whether oxycodone plays a protective role in the mifepristone‐induced injury model by activating the Keap1/Nrf2/HO‐1 signaling pathway. Mifepristone‐induced hEndoSCs were treated with oxycodone (10 μg/mL) or/and a Keap1/Nrf2/HO‐1 inhibitor (5 μM ML385) for 24 h. We observed that oxycodone enhanced the expression of Keap1, Nrf2, HO‐1, and NQO1 in mifepristone‐treated hEndoSCs (Figure 3A–E). The same trends were observed in the RT‐qPCR analysis (Figure 3F–I). However, these effects were reversed by treatment with ML385. Our findings suggest that ML385 reverses the effects of oxycodone on the Keap1/Nrf2/HO‐1 signaling pathway.

### 3.6 ML385 reversed the effects of oxycodone on cell viability, cell apoptosis, and caspase3 activity in hEndoSCs

**Figure 4:**
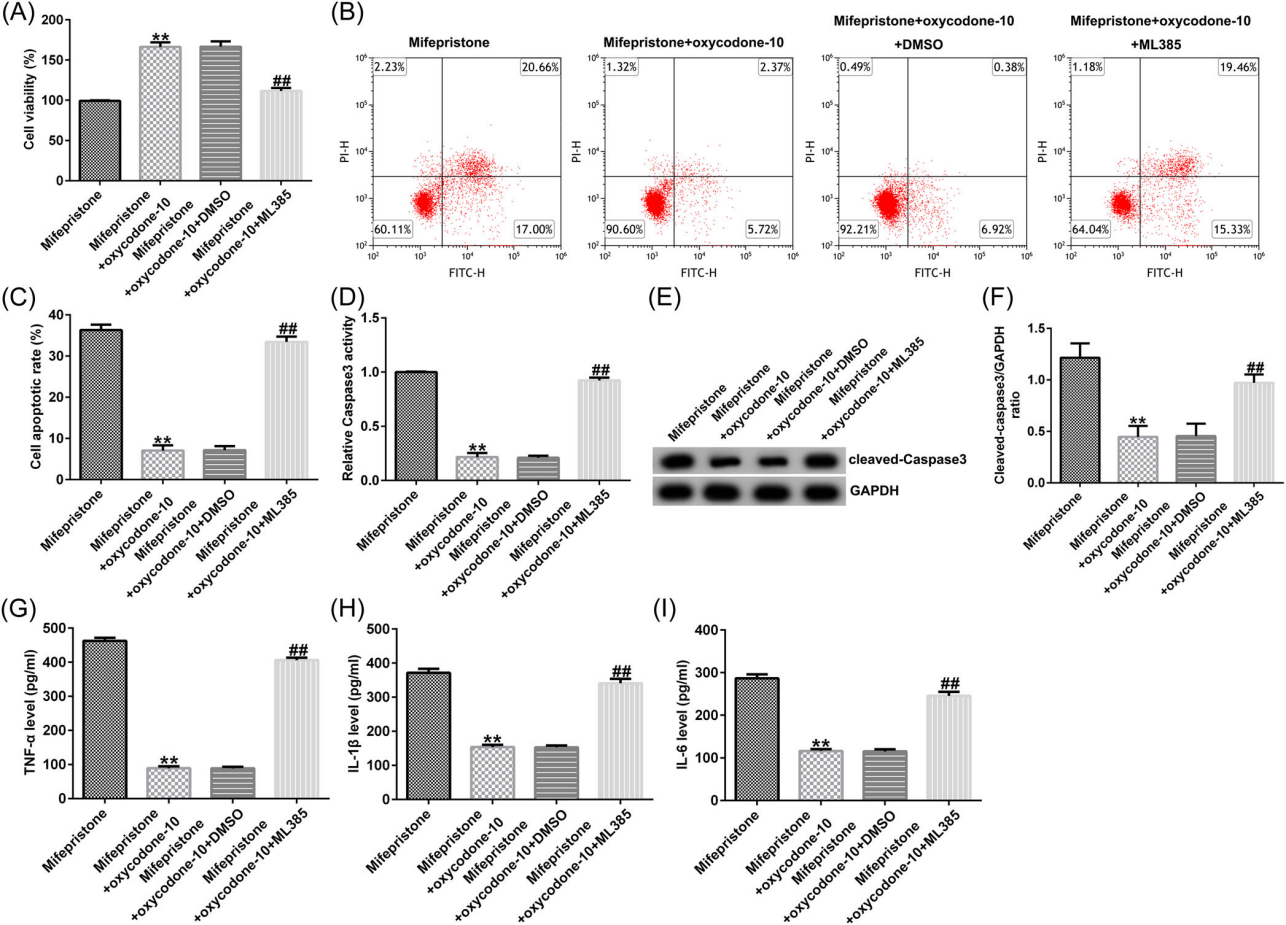
ML385 reversed the effects of oxycodone on cell viability, cell apoptosis, and inflammatory response in mifepristone‐treated human endometrial stromal cells (hEndoSCs). hEndoSCs were exposed to mifepristone for 48 h, and then treated with oxycodone (10 μg/mL) and/or a Keap1/Nrf2/HO‐1 inhibitor (5 μM ML385) for 24 h. Cells were divided into four groups: mifepristone, mifepristone +  oxycodone‐10, mifepristone + oxycodone‐10 + DMSO, or mifepristone + oxycodone‐10 + ML385. (A) hEndoSC viability was evaluated using the MTT assay. (B) hEndoSC apoptosis was evaluated using flow cytometry. (C) Quantification of apoptotic cells. (D) Determination of Caspase3 activity. (E) Western blot analysis of cleaved‐Caspase3. (F) Quantification of cleaved‐Caspase3 expression. The enzyme‐linked immunosorbent assay was applied to assess tumor necrosis factor‐α (TNF‐α), interleukin‐β (IL‐β), and IL‐6 (G–I) expression in the different groups. *N* = 3. **p < .01 versus mifepristone; ^##^ *p* < .01 versus the mifepristone + oxycodone‐10 + DMSO group. Experiments were repeated three times, and the data were presented as means ± SD.

Next, we aimed to determine whether oxycodone plays a role in cell viability, apoptosis, and caspase3 activity in mifepristone‐induced hEndoSCs through the Keap1/Nrf2/HO‐1 signaling pathway. hEndoSCs were exposed to mifepristone for 48 h, and then treated with oxycodone (10 μg/mL) or/and a Keap1/Nrf2/HO‐1 inhibitor (5 μM ML385) for 24 h. Our results suggested that ML385 reversed the effects of oxycodone on cell viability, cell apoptosis, and caspase3 activity in hEndoSCs, as evidenced by the decreased cell viability (Figure 4A), increased apoptotic cells (Figure 4B,C), and enhanced Caspase3 activity (Figure 4D) and cleaved‐Caspase3 expression (Figure 4E,F). Our findings suggest that oxycodone reverses the effects of mifepristone on cell viability, apoptosis, and the activity of Caspase3 in hEndoSCs by activating the Keap1/Nrf2/HO‐1 signaling pathway.

### 3.7 ML385 reversed the effects of oxycodone on the production of inflammatory cytokines in hEndoSCs

Finally, we investigated whether oxycodone exerted effects on inflammatory responses in mifepristone‐induced hEndoSCs via the Keap1/Nrf2/HO‐1 signaling pathway. Our data indicated that oxycodone suppressed the release of TNF‐α, IL‐β, and IL‐6 in mifepristone‐induced hEndoSCs (Figure 4G–I), while this inhibition was at least partly reversed by ML385 treatment. Collectively, our findings indicate that the Keap1/Nrf2/HO‐1 signaling pathway is associated with mifepristone‐treated hEndoSCs and that oxycodone plays a protective role in mifepristone‐induced hEndoSC injury by activating the Keap1/Nrf2/HO‐1 signaling pathway.

## 4 DISCUSSION

Oxycodone, which is a semisynthetic opioid, has been used as a powerful analgesic for over 80 years,^25^ and is widely used in clinical practice due to its high bioavailability and multiple drug delivery routes.^26^ Yang et al.^27^ revealed the protective effects of oxycodone on neuropathic pain in mice. Endometrial injury is a common gynecological disease caused by a variety of factors, including endocrine disorders, inflammation, and abortion, and in severe cases, may cause infertility.^1^, ^28^ Our previous research found that oxycodone alleviates endometrial injury via the inhibition of the activation of the TLR4/NF‐kB pathway.^11^ However, the function of oxycodone and its latent pathogenesis in endometrial injury have not been completely investigated. Thus, it is crucial to elucidate the possible mechanisms of oxycodone and investigate effective treatments for endometrial injury.

Previous studies have revealed that mifepristone, curettage, and coagulation are widely used to stimulate cells to generate endometrial injury models.^24^, ^29^ Mifepristone is clinically used for early pregnancy, promoting menstruation, stopping pregnancy, and certain gynecological surgeries, including the placement and removal of intrauterine devices, laser separation of abnormal cervical tube development, and cervical dilation and curettage.^30^ hEndoSCs play a key role in repairing endometrial injury,^19^ and endometrial injury leads to hEndoSC apoptosis and reduction of hEndoSC viability.^20^ Mifepristone‐induced hEndoSC injury has been used to study endometrial injury in vitro.^11^, ^23^, ^24^ In our study, hEndoSCs were treated with mifepristone to generate an in vitro endometrial injury model. Consistent with previous studies,^11^, ^23^, ^24^ the mifepristone‐induced hEndoSC injury was successfully established in this study, evidenced by reduced cell viability, enhanced cell apoptosis, and inflammatory response.

To assess the functions of oxycodone in mifepristone‐induced endometrial injury, hEndoSCs were treated with 1, 5, 10, 15, or 20 μg/mL oxycodone.^11^ We first verified that oxycodone had no cytotoxic effect on hEndoSCs.^11^ To better illustrate the roles of oxycodone in mifepristone‐induced endometrial injury, hEndoSCs were treated with mifepristone and 1, 5, or 10 μg/mL oxycodone. Our data revealed the protective effect of oxycodone against mifepristone‐induced endometrial damage, as evidenced by the increased cell viability and inhibition of apoptosis.

It is generally believed that caspase3 is the most important terminal shear enzyme during apoptosis and an important part of the cytotoxic T lymphocyte cell‐killing mechanism.^31^, ^32^ Therefore, we also investigated the Caspase3 activity and cleaved‐Caspase3 levels in oxycodone‐treated hEndoSCs. We found that oxycodone exposure markedly reduced Caspase3 activity and cleaved‐Caspase3 expression levels. Previous studies have revealed that inflammatory factors, such as TNF‐α, IL‐β, and IL‐6, contribute to endometrial injury.^1^ Also, administration of mifepristone could induce the section of inflammatory cytokines (such as TNF‐α, IL‐β, or IL‐6).^11^, ^33^ Thus, the levels of TNF‐α, IL‐β, and IL‐6 in hEndoSCs were detected, and consistent with the previous study,^11^ the findings indicated that mifepristone promoted TNF‐α, IL‐β, and IL‐6 secretion in hEndoSCs. Oxycodone reduced inflammatory cytokine release in hEndoSCs in a dose‐dependent manner, indicating that oxycodone abolished inflammatory cytokine release during mifepristone‐triggered hEndoSC damage. However, it should be noted that a full spectrum of the cytokine array other than TNF‐a, IL‐1β, and IL‐6 will make our investigation more convincing.

It has been reported that multiple pathways, including the ROS/NLRP3/caspase‐1/GSDMD, WNT, and mTOR pathways, are associated with the pathogenesis of endometrial injury.^12^, ^13^, ^14^ The Keap1/Nrf2/HO‐1 signaling pathway, a classic pathway related to oxidative stress, has been reported to be involved in many diseases, including osteoclastogenesis,^34^ type 1 diabetes,^35^ and nephrotoxicity.^36^ In recent years, increasingly more studies have focused on the correlation of the Keap1/Nrf2/HO‐1 signaling pathway with inflammatory response.^17^, ^37^, ^38^ In addition, a recent study found that the Keap1/Nrf2/HO‐1 signaling pathway is involved in LPS‐induced epithelial cell inflammatory damage.^39^ However, it is still unclear whether the Keap1/Nrf2/HO‐1 signaling pathway is involved in mifepristone‐induced inflammatory damage to endometrial stromal cells, and whether oxycodone can affect the activation of the Keap1/Nrf2/HO‐1 signaling pathway. As expected, in this study, mifepristone treatment significantly inhibited the Keap1/Nrf2/HO‐1 signaling pathway, evidenced by the decreased levels of Keap1, Nrf2, HO‐1, and NQO1.^40^ However, oxycodone treatment activated the Keap1/Nrf2/HO‐1 signaling pathway in mifepristone‐induced hEndoSCs. To better illustrate the latent mechanism of oxycodone in a mifepristone‐induced endometrial injury model, a Keap1/Nrf2/HO‐1 pathway inhibitor (ML385) was used.^41^ Further functional analysis revealed that ML385 reversed the effects of oxycodone on mifepristone‐treated hEndoSCs by deactivating the Keap1/Nrf2/HO‐1 signaling pathway, as confirmed by the inhibition of Keap1, Nrf2, HO‐1, and NQO1 expression, suppressed cell viability, and promotion of apoptosis, Caspase3 activity, cleaved‐Caspase3 expression, and inflammatory cytokine release, suggesting that the protective effects of oxycodone may be regulated, in part, through the Keap1/Nrf2/HO‐1 signaling pathway.

It should be noted that this study also has a few limitations. First, this study was not conducted in the primary cell cultures of endometrial stromal cells, which is a limitation of this study, and a co‐culture model of endometrial injury using epithelial and stromal fraction will make this study more convincing. Besides, the molecular mechanism involved in activating the Keap1/Nrf2/HO‐1 signaling pathway by oxycodone still needs to be investigated further. Time dependency of oxycodone treatment was also not investigated in this study. In addition, we did not examine the impact of ML385 alone on mifepristone‐induced hEndoSC injury, and this was also a limitation of this study. We will focus on resolving these issues in the future.

In summary, our study provides new insights into the latent mechanism by which oxycodone alleviates mifepristone‐induced hEndoSC injury by activating the Keap1/Nrf2/HO‐1 signaling pathway, suggesting the protective effect of oxycodone on endometrial injury. This study provides a theoretical basis for the clinical treatment of endometrial injury using oxycodone. Oxycodone may be a candidate for combination therapy for treating endometrial injury.

## AUTHOR CONTRIBUTIONS

**Aibing Zhu**: Conceptualization; data curation; formal analysis; investigation; visualization; writing—original draft; writing—review & editing. **Fei Yao**: Software; validation; visualization. **Mingkun Shen**: Investigation; methodology; project administration; resources; writing—review & editing.

## CONFLICT OF INTEREST STATEMENT

The authors declare no conflict of interest.

## Supporting information


**Supporting Information**.

## Data Availability

The data sets used and/or analyzed during the current study are available from the corresponding author upon reasonable request.
