# Supplementary figures and images for: Oxycodone alleviates mifepristone‐stimulated human endometrial stromal cell injury by activating the Keap1/Nrf2/HO‐1 signaling pathway
Source: Immun Inflamm Dis. 2023 Sep 20;11(9):e1008. doi: 10.1002/iid3.1008 (PMC10510466; doi:10.1002/iid3.1008)

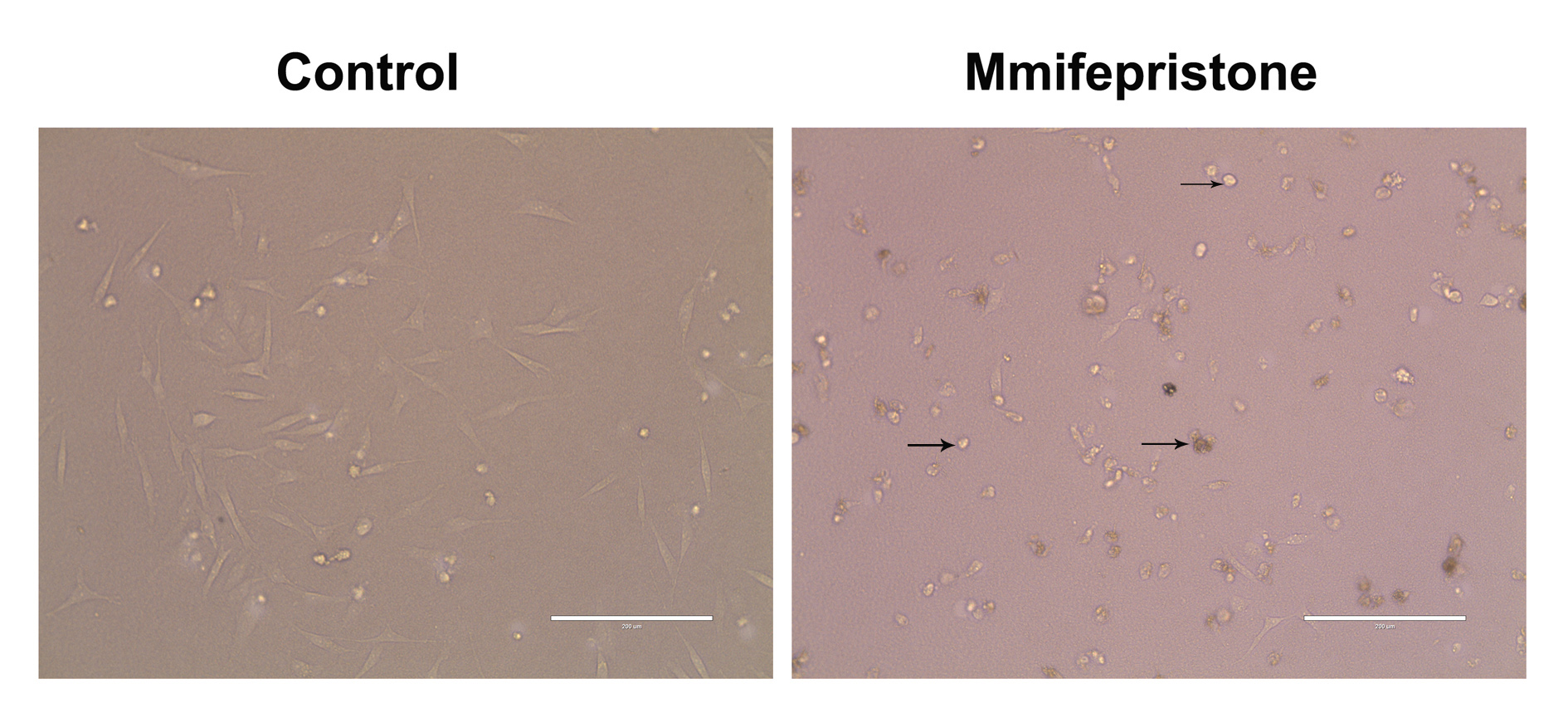

Supplement: Supplementary file 1 — Supporting Information. [file IID3-11-e1008-s001.jpg]
